# Explaining the challenges of Iranian caregivers in provision of home health care to spinal cord injury patients: a qualitative study

**DOI:** 10.1186/s12912-024-01797-0

**Published:** 2024-02-29

**Authors:** Nasrin Galehdar, Heshmatolah Heydari

**Affiliations:** 1 Social Determinates of Health Research Center, Lorestan University of Medical Sciences, Khorramabad, Iran; 2 Social Determinants of Health Research Center, School of Nursing and Midwifery, Lorestan University of Medical Sciences, Khorramabad, Iran; 3 French Institute of Research and High Education (IFRES-INT), Paris, France

**Keywords:** Home health care, Spinal cord injury, Caregiver

## Abstract

**Background:**

The incidence of spinal cord injury (SCI) is increasing across the globe. The caregivers of patients with spinal cord injuries experience many problems during providing care to these patients. Identifying the problems experienced by caregivers can facilitate the process of care provision to these patients. So, the aim of this study was to explore the challenges of caregivers in provision of home health care to SCI patients.

**Methods:**

This study was conducted by qualitative description approach in Iran from Apr 2021 to Dec 2022. The participants included the caregivers of SCI patients recruited by purposive sampling. The data were collected by face-to-face interviews and analyzed using the method proposed by Lundman and Graneheim.

**Results:**

Two themes emerged from the data analysis, including burnout (with the categories of physical challenge and psychological challenges) and coping strategies (with the categories of social support and professional support).

**Conclusion:**

Resolving the obstacles and problems faced by home caregivers can improve the circumstances of care provision so that they can be relieved of their own physical and psychological conundrums and deliver suitable home care to SCI patients.

**Trial registration number:**

Not applicable.

## Background

Spinal cord injury is defined as damage to the spinal cord (SC) that permanently or temporarily causes changes in its function and divided into traumatic and non-traumatic [1]. Today, the prevalence and incidence of SCI is increasing, reaching 20.6 million people and 0.9 million annually, respectively [2]. The prevalence rate of traumatic SCI in Iran is 3 per 100,000 people; its incidence rate is, 10.5 per million people [3].

Based on the anatomical location of the lesion, spinal cord injury can be classified into cervical, thoracic, lumbar and sacral levels. The severity and types of spinal cord injuries could depend on the part of the spine that is damaged [1].

SCI ensues with various disorders in the body’s systems such as the musculoskeletal, sensory-motor, gastrointestinal, urinary, respiratory systems, cardiovascular and integumentary systems besides psychological problems in patients [4, 5]. Each of these disorders impose a high burden on the patient, family and the health system [6], requiring spending a great amount of money by the individual and the society [7].

In addition, following the acute period of the disease, the individual may become dependent entirely or partially, requiring receiving care for years [6]. There are rehabilitation centers all over the world where both new technologies and expert staff are brought together to provide the highest level of patient care [8].

Management of chronic diseases, such as SCI, is among the main challenges of the health system, and home care offers a leading strategy to reduce the complications and duration of hospitalization [9].

Professional home care is a type of care that persons with SCI received services in their home from a health care professional such as a nurse [10]. In the home care services, the difference between professional and informal care is unclear. The finding of a study showed that the presence of professional caregivers at home can be effective in supporting of informal caregivers SCI patients, but it is not a replacement [11]. In this path, family’ caregivers face numerous issues and problems caused by factors such as the lack of educational, supervisory, and specialized systems, which can facilitate transition from hospital-based care to maintenance home care, as one of the important pillars of the health care chain of chronic diseases [12]. Professional caregivers can play a vital role in preparing the family to continue caring for the patient.

There are few insights on the use of professional home care services for persons with SCI. Caring of persons with SCI require to collaboration between professional and informal caregivers [13]. The few international studies accessible examined the effect of professional home care services on outcomes of SCI patients such as hospitalization and emergency department use [11–13], or cost [13, 14]. Studies conducted on elderly European populations showed higher use of professional home care can lead to increased use of informal care [15, 16], but other studies showed that the use of professional home care led to a decrease in the use of informal care [17, 18].

Iran’s health system is structured on three levels of healthcare and referral, and health services is provided based on the primary health care (PHC) approach. According to this structure, urban and rural health centers provide health services to the population of that neighborhood [16].

Home-based services for SCI patients not to provide in this structure, and private, charity centers and the state welfare organization (Behzisti Organization) provide home-based care to this patients [19]. Private home care centers are set up under the supervision of the Ministry of Health. Various professionals such as physician, nurses and physiotherapists are provided care, cure and rehabilitation services to clients in these centers [12].

Family caregivers bear the main role in home care provision, and this capacity can help formal caregivers guarantee the continuation of the patient’s care. Professional caregiver in the general home care services maybe experience issues such as workload, in Lack of security, insufficient wages, lack of insurance coverage, lack of family cooperation, lack of support from officials [20]. Home care for persons with SCI is different of other patients, and caregivers maybe experience other problems. So far, few studies have been conducted to explaining the challenges of professional caregivers of SCI patients in Iran. Identifying and resolving caregivers’ problems can help upgrade the level of the care provided. Considering that limitation information in this field, it seems that conducting a study by qualitative method could be helpful in identifying of professional caregivers’ problems. So, this study was designed with the aim of explore the challenges of Iranian caregivers in provision of home health care to spinal cord injury patients.

## Methods

This study was conducted by qualitative description approach [21] in Khorramabad city, Iran from Apr 2021 to Dec 2022.

### Participants

Participants in this study were selected by purposive sampling and included formal caregivers engaged in care provision to SCI patients. In this study, Professional caregivers were people who had received formal training for caring of persons with SCI and had received a valid certificate or were nursing graduates. The inclusion criteria for them encompassed being involved in providing home care to SCI patients for at least one year, affiliation to the health care provider centers (Behzisti, Private home care or charity center), willingness to participate in the study, and ability to accomplish an interview. Exclusion criteria encompassed unwillingness to participate in the study.

The researcher referred to comprehensive health centers, private home care centers and SCI association to obtain access to participants. The first interview was conducted with an experienced professional home caregiver, and according to the information gathered, the sampling process continued in other locations such as the comprehensive health centers, association support of SCI patients and SCI patients’ home in the form of a snowball. All of interviews were held in the quiet room in the workplace of participants. It was tried to meet maximal diversity among the participants in terms of age, sex and experience.

### Data collection

In this study, the data were collected by conducting 10 face-to-face semi-structured interviews (Table 1). The researcher founded a close and direct relationship with the participants in order to reach authentic and true information.

The general question guiding the interviews was: How do you describe your experience of home care? What are the challenges of taking care of SCI patients at home? The researcher directed the interview to achieve prespecified goals using probing questions. It is noteworthy that the main question of the study was slightly modified during the research based on the data collected and the objective of the study. Sampling continued until data saturation was reached, this means that no new information was obtained in the subsequent interviews [22]. The duration of each interview was between 15 and 40 min, and all interviews were recorded using an electronic device.

### Data analysis

In this study, two authors were involved in the analytical process. The data analysis proceeded simultaneously with the interviews using the method proposed by Graneheim and Lundman’s content analysis approach [23]. The interviews were transcribed verbatim immediately after each interview. In order to arrive an overall understanding of the content, the interviews were listened to several times and their transcripts were read several times. The whole interview was considered as the unit of analysis and then the words, sentences and paragraphs formed meaning units. Meaning units were considered as code. Then the codes were sorted in more abstract categories based on their similarities and differences. After merging the subcategories and then categorizes together, the concepts latent in the data were emerged that labeled as themes.

### Trustworthiness

Authors used The Lincoln and Guba’s criteria including credibility, dependability, confirmability and transferability for keeping the trustworthiness of the study findings [24].

The researchers were familiar with qualitative research and were engaged in providing community-based services to patients for many years.

For credibility, the researcher spending sufficient time in the field, which helped to understand the culture, social setting and developing relationships with members or organizations which provide community-based services to SCI’ patients. After the identification of primary codes, participants’ opinions were sought to ensure the accuracy and interpretations of the codes, and if any inconsistency was detected between participants’ opinions and the codes extracted, necessary amendments were considered. Two experts (one college professor and one specialist in the field of qualitative research) were requested to express their opinions on the selected codes and categories. Also, it was tried to enroll participants with maximal diversity in terms of knowledge, experience, duration of service, workplaces during service, age, and gender. For transferability, authors tried report the study with describing details of research context and assumptions.

### Ethics approval and consent to participate

All methods were performed in accordance with the relevant guidelines and regulations of the declaration of Helsinki (ethical approval and consent to participate). The aims and methods of the project were explained to all participants, and necessary assurance was given to them about the anonymity and confidentiality of their information and audio files. Informed consent was taken from all participations. The participants had the right to withdraw of study during or at any other time. The study approval was taken from the ethics committee of Lorestan University of Medical Sciences ethics code of IR.LUMS.REC.1395.192.

## Results

In this study, the data were gathered by conducting 10 face-to-face interviews (Table 1). Data analysis led to the emergence of 683 initial codes, which were classified into two themes, including burnout and coping strategies (Table 2).

**Table 1:** Participants’ characteristics

Participant’s code	Sex	Age	Level of education	Experience (years)	Marital status	Type of caregiver
1	Female	31	Post-graduate	7	Married	Formal
2	Male	27	Associate Degree	6	Married	Formal
3	Male	29	Bachelor	1	single	Formal
4	Male	32	Bachelor	10	Married	Formal
5	Male	38	Post-graduate	10	Married	Formal
6	Male	32	Post-graduate student	1	single	Formal
7	Female	32	Bachelor	6	Single	Formal
8	Male	30	Bachelor	6	Single	Formal
9	Male	34	Post-graduate student	6	Married	Formal
10	Female	30	Bachelor	8	Married	Formal

**Table 2:** The categories and sub-categories extracted after data analysis

Theme	Categories	Sub-Categories
Burnout	Physical challenges	Caregivers’ workload
		Risk of musculoskeletal complications
	Psychological challenges	The loss of the concept of time
		The continues of care provision
		Non-acceptance of the caregiver by the patient and the patient’s family
		Caregivers’ lack of motivation
		Difficulty in establishing a relationship between caregivers and patients’ families
Coping strategies	Social Support	Necessity of pension insurance
		The necessity of liability insurance
		Financial challenge
	Professional Support	The need for empowerment
		Adapting the caregiver to clients

## Burnout

According to data analysis, SCI patients need numerous care requirements, causing caregivers to develop chronic fatigue during the care process. Caregivers may also face life attrition due to chronic physical and mental pressure. Under this theme, there were two categories, including physical and psychological challenges.

### Physical challenges

Data analysis showed that the caregivers of SCI patients are affected by physical issues during the care process. SCI patients have many care needs during the day. The caregivers experience excessive issues due to prolonged and continuous provision of such services. Under this category, two subcategories were identified: caregivers’ workload and the risk of musculoskeletal complications.

#### Caregivers’ workload

Data analysis revealed the high workload of caregivers, causing them to develop attrition during their work. SCI patients are unable to perform their usual tasks such as sleeping, walking, sitting, eating, going to the toilet, showering and…, many of which need to be taken care of by the caregiver. Caregivers are actively involved in bathing, feeding, changing diapers and repositioning their patient. One participant noted the effects of heavy workload on his power: “…I lost more than 10 Kg within one year of working. I used to weigh 83 Kg, and currently I am 71 Kg…” [6].

#### Risk of musculoskeletal complications

According to finding of this study, moving and changing the position of heavy patients over time may harm caregivers’ musculoskeletal system. Some of musculoskeletal problem can including spine deviation such as scoliosis, lordosis, kyphosis, disk herniation and osteoarthritis. One of the participants addressed this problem as: “…my client was an athlete and had strong muscles and sturdy bones…it was difficult to translocate him…” [4].

### Psychological challenges

The data showed that the caregivers endured psychological pressure. An unpleasant atmosphere in the environment where care is provided, lack of social status, lack of ranking among caregivers, and continuous care provision can lead to demotivation and psychological exhaustion among caregivers. Four subcategories, including the loss of the concept of time, the continues of care provision, non-acceptance of the caregiver by the patient and the patient’s family, caregivers’ lack of motivation, and difficulty in establishing a relationship between caregivers and patients’ families, were discovered under this category.

#### The loss of the concept of time

Data analysis revealed that caregivers allocated a lot of time to provide home care to the patient. The participants stated that their services were delivered round the clock and might be extended seven days a week. In this regard, one of the participants noted: “…sometimes, a single day was like 10 days and even one week to me; time had a different meaning to me, it passed by slowly…” [5].

#### Continues of care provision

Data analysis showed that the continuity of care provision, constant need for moving the patient, and ambiguity in the patient’s recovery process contributed to psychological exhaustion of caregivers. One of the participants addressed the negative impacts of the governing atmosphere on the environment: “…I used to start every morning with sighs, wails, and complaining from pain and end it in the same manner…” [5].

#### Non-acceptance of the caregiver by the patient and the patient’s family

According to data analysis, one of the problems of the caregivers was non-acceptance by the patient and his/her family. Moreover, the data showed that caregivers might receive unkindness from the patient and the family, eventually disheartening the caregiver and leading him/her to quit the job. In this regard, one of the participants noted: “…In my opinion, another challenge is the lack of cooperation of the patient with the caregiver; they become stubborn frequently, refusing the task they have been requested to do… “ [6]. Another participant mentioned: “…They sometimes get angry and behave rudely towards the caregiver…. bad attitudes discourage caregivers and cause them to quit the job….” [4].

#### Caregivers’ lack of motivation

Data analysis showed that there are no encouragement policies for caregivers. In fact, there is no discernment between those who strive to improve the client’s condition and his/her quality of life and those who make less effort. Caregivers are not ranked based on the quality of the care delivered and the outcomes targeted by the health system. The data indicated the necessity of offering motivations to caregivers to make them stay in the job and provide better services. Also, the quality of services provided by them should be taken into account. This issue was addressed by one of the participants as: “… it is necessary to offer job motivations to create a positive competitive atmosphere among caregivers so that they more willingly deliver better care services to patients…” [7].

#### Difficulty in establishing a relationship between caregivers and patients’ families

Data analysis revealed that the culture and prevailing atmosphere in a family were serious obstacles hindering caregivers from entering patients’ homes. The prevailing culture and atmosphere in the family may contradict the caregiver’s way of thinking, agonizing his/her psychologically. One of the female caregivers addressed this issue as: “…It is very difficult to establish a relationship with the patient’s family. When a client is introduced to a caregiver, he/she is required to enter into a family’s environment where there may be distinct cultural values, moral standards, and certain prejudices…” [1].

## Coping strategies

One of the problems of caregivers is the difficulty in cope with the caregiving situation. Various external and internal factors can help in creating this compatibility. When caregivers are able to meet the needs of the patient and family well, they are better able to adapt to their caregiving profession. Under this theme, there were two categories, including social support and professional support.

### >Social support

Social support defined as a person’s access to a supportive social network and help from other people. Social support can comprise psychological, spiritual and financial support for caregivers so that they can exploit their capabilities to provide care for patients. These supports can help to caregivers for adjustment with their role. Under this category, there were three subcategories, including the necessity of pension insurance, the necessity of liability insurance and financial challenge.

#### The necessity of pension insurance

Data analysis highlighted that caregivers who provide care to patients should receive support from social institutions. Spending their time taking care of patients, caregivers lose their golden time and opportunity to be hired, have income, and develop skills. Therefore, caregivers should be covered by supporting organizations. In this regard, one of the participants mentioned: “…. I was nursing for ten years and because of that I could not get a job… my job was to take care of patients…wherever I looked for a job, they told me that I was too old and asked why I remained jobless for so long….” [5].

#### The necessity of liability insurance

Data analysis showed that one of the problems of the caregivers of SCI patients was the fact that they lacked any insurance, depriving them from any support by anyone if they encountered a problem during care provision to the patient. Addressing caregivers’ lack of having liability insurance, one of the participants stated: “… this means that if a caregiver faces any problem during work…. for example, when I lift a patient with SCI, if he/she falls on my hand and broke it, or if anything happens to me, there is no liability insurance to support me…. on the other hand, if anything happens to the patient, insurance does support the caregiver…” [4]. The same participant continued sharing his experience: “…I had a client who had been on the bed for 12 years…. during taking care of him, his arm broke…. the authorities said to me that it has nothing to do with us, you should be pay the compensation…” [4].

#### Financial challenge

According to data analysis, the caregivers are paid low wages, and considering the nature of their job, these insufficient payments can negatively affect their motivation for working. One of the caregivers described this issue as: “…we are paid a minimal wage…it is only sufficient for buying food so one does not die…” [4].

#### Professional support

One of the needs of SCI patients is to improve the quality of their care. Caregivers should be receiving the necessary training to meet the needs of patients. Also, formal caregivers should be selected based on needs of patients and their ability to meet these needs, for be able to provide quality care to patients. Under this category, there were two subcategories, including the need for empowerment and the necessity of matching the patient with the caregiver.

#### The need for empowerment

Data analysis showed that caregivers needed empowerment to be able to support and provide care to SCI patients. The participants noted that in many cases, family members lacked the required skills on how to move patients, fulfill their physiological needs, and prevent psychological and physical complications, so they needed to learn such skills in educational classes. In this connection, one of the caregivers expressed: “…. there is a need for educating communication skills, what kind of communication should be established when the patient behaves angrily… knowing that the caregiver should not reciprocate…training on how to employ occupational therapy or how to use the physiotherapy machine…” [5]. Another participant mentions noted: “…these caregivers receive no training in the hospital at all…. education on what tasks they need to do at home, for example, to be able to better take care of the patient and to avoid the patient from developing bed sores or infectious diseases and to enable him to exploit his/her abilities more easily…” [8].

#### Adapting the caregiver to clients

Caregivers should be selected considering criteria such as the patient’s physical limitations, distance, living place, and the cultural status of the family. Accordingly, one of the female caregivers stated: “…the family reiterated that a female caregiver should come to take care of their patient, why? Because the environment was feminine, her sister and her mother were there…”. Another participant underlined the need for the caregiver’s being sufficiently strong to move the patient: “…when you have to deal with heavy muscles and to lift a patient…. these cannot be done by a female for a male client…” [4].

## Discussion

The analysis of the data showed that caregivers while providing care to SCI patients may gradually develop numerous physical and psychological problems secondary to the type and continuity of the services delivered by them. In order to minimize these problems, these caregivers need to be professionally supported by the system.

According to data analysis, one of the problems of caregivers was job burnout over time. Consistent with the findings of this study, the observations of other studies have shown that caregivers experience high levels of care burden, depression, anxiety, physical exhaustion, reduction in life satisfaction, social isolation, and identity loss [25, 26]. This burden of care can be related to the fear of the patient’s uncertain fate, the patient’s dependence, the caregiver’s excessive fatigue, and financial issues. Also, the demographic features of the patient and the caregiver can influence the amount of care burden [27].

In this study, it was specified that one of the factors contributing to job burnout in caregivers was constant care provision for many years, which can gradually wear away caregivers’ mind and body. In line with the findings of this study, other studies’ results have indicated that, in contrast to care provision in other conditions, caregiving in SCI patients may last several decades because these patients now enjoy an increase in life expectancy. On the other hand, caregivers often accept this role in the early middle-age period [25, 28]. On the contrary, the results of another study suggested that care burden was not significantly associated with the duration of the injury or caregiving period, highlighting caregivers’ ability to adapt themselves to new roles, responsibilities, and situations, and problems over time [27]. The patient’s dependence on the caregiver, the caregiver’s responsiveness, and personal characteristics, as well as social, financial, and demographic parameters can greatly affect the extent of care burden [27, 29–31].

Physical pressure and fatigue experienced by caregivers are among the factors that can contribute to job burnout in these individuals. In line with our findings, the results of another study demonstrated caregivers endured enormous physical pressure when they had to move patients for taking a shower or using the bathroom [27]. Factors such as age [32–34], gender [29, 30, 35] literacy level, occupation [36, 37], patients’ physical activity [38], economic status, and social support [39] can influence the care burden imposed on caregivers. Also, variabilities in people’s personality in terms of resilience and behavioral attitudes, as well as demographic, economic, and geographical parameters can significantly affect the level of care burden experienced by caregivers [29, 32, 39]. Assistive technologies enable individuals with disabilities live independent and healthy and their care of them is facilitated [40]. The use of these technologies for patients with SCI is not common in Iran [19], so it is necessary to make these technologies available to patients and families.

The findings of the present study showed that the caregivers experienced numerous psychological problems. In line with this finding, the observations of another study also indicated the high frequency of psychological problems among caregivers. It was reported that young and educated caregivers experienced low care burden because they could easily understand the patient’s needs and problems, successfully manage stressful situations, and appropriately adapt to their new roles by exploiting suitable coping strategies [27].

Findings of this study highlighted professional support as one of the important needs of caregivers of SCI patients, and the fact that these individuals should receive psychosocial and spiritual support from supporting organizations. In line with the findings of this study, the findings of the other studies showed that family caregivers have many emotional, social and informational needs that should be supported [41, 42]. The results of other studies indicated that caregivers’ job influenced their professional, social, and familial relationships [43, 44].

Because caregivers need to accept new roles besides the duties assigned to them already, they may face problems such as difficulty in adapting to the new role and also the necessity of acquiring different and complicated skills [45]. Therefore, supporting organizations need to consider the necessary measures to help these people adjust themselves with their new roles and learn the new skills required. On the other hand, since home caregivers require to take care of a disabled individual for many years, they may lose the opportunity of being employed or learning job skills, so it is necessary to provide these people with monthly salaries and benefits from retirement.

The findings of this study indicated that the level of the SCI should be proportionate to the type of caregiving. In parallel with this observation, the results of another study showed that the type and level of the SCI could affect caregivers’ physical functioning and life quality [46].

One of the important limitations of this study was conducting the interviews during the Covid-19 pandemic period. Considering that SCI patients were part of the vulnerable population in terms of contracting covid-19, some caregivers did not willing participate in the interview. Therefore, it was tried to collect data by following the health protocols and making sure that the interviewers are not sick. To ensure the health of patients and their families, a complete subjective and observative assessment including interview, fever control and rapid COVID-19 test was performed.

## Conclusion

While taking care of patients with SCI, caregivers develop numerous physical and psychological problems due to the type of the work and prolonged service provision. In order to minimize these problems, it is necessary that these caregivers receive professional support from supporting organizations and benefit from employment advantages such as pension insurance and liability insurance. Considering the type of patients, caregivers should be appropriately empowered to be able to deliver the services that the system and the client expect from them to fulfill. Considering that a limited number of studies have been conducted in this field, more studies are required to be performed in future.

## Data Availability

The datasets generated during and/or analyzed during the current study are available from the corresponding author on request.

## Abbreviations

SCI: Spinal cord injury
SC: Spinal Cord
PHC: Primary health care
